# P08-03 Integrating public health expertise to support green space planning by promoting active lifestyles in Slovenia

**DOI:** 10.1093/eurpub/ckac095.116

**Published:** 2022-08-29

**Authors:** Ina Šuklje Erjavec, Andrea Backović Juričan, Jana Kozamernik, Tjaša Knific

**Affiliations:** 1 Urban Planning Institute of the Republic of Slovenia, Ljubljana, Slovenia; 2 Prevention and Promotion Programmes Management, National Institute of Public Health, Ljubljana, Slovenia

**Keywords:** expert collaboration, green spaces, spatial planning, HEPA, municipality guidelines

## Abstract

**Issue/problem:**

To minimize public health risks and promote HEPA among citizens, an integrated approach between public health experts and spatial planning is much needed. However, cross-sectoral cooperation is very demanding and needs harmonization of concepts and professional terms to reach mutual understanding.

**Description of the problem:**

In 2017 the Urban Planning Institute of the Republic of Slovenia launched the programme entitled Expert Basis for Spatial Planning of Green Areas, aimed at HEPA promotion for citizens. It was co-financed by the Ministry of Health and based on the objectives in the National Programme on Nutrition and Health Enhancing Physical Activity 2015-2025 and the Strategy of the Government of the Republic of Slovenia for Children and Youth Environmental Health 2012-2020. Its purpose is to expertly support the planning and development of towns/settlements in Slovenia for better quality of life and direct it for active and healthy lifestyles of citizens of all ages and social groups. To properly address all relevant issues, cooperation with the National Institute of Public Health was established.

**Results (effects/changes):**

The programme contained multidisciplinary collaboration in a form of consultation workshops and an expert review of the programme publication named Going Out for Health: A green space planning manual to promote physical activity and a healthy lifestyle. The emphasis was on supporting spatial planning issues on quality of green spaces with basic objectives of public health perspectives. The guide is the first example of linking public health promotion approaches and quality aspects of green space planning and design in Slovenia. It contains general guidelines for the planning of green areas aimed at promoting HEPA, expert materials to support municipalities in devising relevant approaches, and decision-making in order to provide useful and attractive green areas.

**Lessons:**

The aspects of green area spatial planning aimed at promoting HEPA are diverse and closely interconnected. The programme and its manual reflect a good cooperation example between spatial planning and public health experts.

**Main messages:**

A good collaboration between urban planners and public health professionals is crucial to prepare useful guidelines for spatial planners and municipality decision-makers on green space planning for HEPA promotion.

